# Relationship Between Prolonged Intraocular Inflammation and Macular Edema After Cataract Surgery

**DOI:** 10.1167/tvst.10.7.15

**Published:** 2021-06-14

**Authors:** Alexander Aaronson, Claudia Taipale, Asaf Achiron, Vesa Aaltonen, Andrzej Grzybowski, Raimo Tuuminen

**Affiliations:** 1 Helsinki Retina Research Group, University of Helsinki, Helsinki, Finland; 2 Department of Ophthalmology, Helsinki University Hospital, Helsinki, Finland; 3 Department of Ophthalmology, The Edith Wolfson Medical Center, Holon, Israel and Sackler School of Medicine, Tel Aviv University, Israel; 4 Department of Ophthalmology, Bristol Eye Hospital, Bristol, UK; 5 Department of Ophthalmology, Turku University Hospital, Turku, Finland; 6 Department of Ophthalmology, University of Turku, Turku, Finland; 7 Department of Ophthalmology, University of Warmia and Mazury, Olsztyn, Poland; 8 Institute for Research in Ophthalmology, Foundation for Ophthalmology Development, Poznan, Poland; 9 Department of Ophthalmology, Kymenlaakso Central Hospital, Kotka, Finland

**Keywords:** aqueous flare, cataract surgery, intraocular inflammation, macular edema, perioperative treatment, pseudophakic cystoid macular edema

## Abstract

**Purpose:**

To assess whether aqueous flare is related to an increased risk of pseudophakic cystoid macular edema (PCME) following uneventful cataract surgery in nondiabetic and diabetic patients.

**Methods:**

A post hoc analysis of five consecutive randomized clinical trials in the Department of Ophthalmology, Kymenlaakso Central Hospital, Finland. Aqueous flare levels were recorded in 448 eyes of 448 patients before surgery, and after the course of topical anti-inflammatory treatment 28 days and three months after cataract surgery.

**Results:**

Aqueous flare increase of <50%, ≥50%, ≥100%, and ≥200% associated in central subfield macular thickness (CSMT) increase across the groups at 28 days and three months after surgery. Increase of aqueous flare ≥100% compared to those with <100% was associated with increased CSMT (*P* = 0.022 at 28 days, and *P* = 0.027 at three months). At three months, macular thickening (at least 10% CSMT increase) was observed in 12.7% compared to 4.6% of eyes when using a cutoff value of 100% increase in aqueous flare (*P* = 0.033). Although diabetic patients presented higher aqueous flare levels at baseline compared to nondiabetic patients (12.9 ± 11.8 vs. 9.8 ± 8.2 photon units/ms *P* < 0.001), the postoperative levels illustrated a similar profile in aqueous flare increase between the two groups.

**Conclusions:**

At 28 days, aqueous flare increase was associated with macular thickening. A 100% cutoff value could potentially be used when studying anti-inflammatory efficacy of different treatment protocols. Flare values exceeding this cutoff value could be considered as an indication for extending anti-inflammatory therapy.

**Translational Relevance:**

A 100% increase in aqueous flare at 28 days after cataract surgery from baseline predicted macular thickening up to three months postoperatively. Identifying a correlation between increased aqueous flare levels and pseudophakic cystoid macular edema may allow recognition of the most vulnerable patients, development of prophylactic treatment strategies and reduction of the number and severity of postoperative complications.

## Introduction

Blood-aqueous-barrier (BAB) disruption and aqueous flare increase are found during a variety of infections, inflammatory diseases, and after ophthalmologic operations. With modern techniques, cataract surgery has a low intraoperative complication rate. However, postsurgical inflammation predisposes eyes to pseudophakic cystoid macular edema (PCME), a late complication that may result in poorer postoperative visual acuity.^1^

The kinetics of aqueous flare, a clinical marker for intraocular inflammation, after cataract surgery were described to peak within the first few postoperative days, after which the levels decline rapidly during the first week and return to the baseline by three months.^2^^–^^4^ Nevertheless, high aqueous flare levels have been associated with multiple proinflammatory and vasoactive cytokines and predicted macular edema after uncomplicated cataract surgery and the development of PCME.^5^^–^^7^ Furthermore, intravitreal concentrations of interleukins and vascular endothelial growth factor (VEGF) remained sustainably elevated in the eyes that had previously undergone uneventful cataract surgery.^8^

A better understanding of BAB disruption, kinetics of intraocular inflammation, and their association to morbidity with pseudophakic eyes may open new horizons in reducing and treating postoperative complications. Laser flare meter provides a noninvasive method for sensitive and repeatable measure of intraocular inflammation and estimation of retinal disease activity.^9^^–^^12^ Flare measurement could aid in the detection of patients at high risk for PCME to guide anti-inflammatory treatment and follow up. Here, we evaluated the changes in aqueous flare after uneventful cataract surgery in relation with macular changes and PCME development in nondiabetic and diabetic patients.

## Materials and Methods

### Study Design

A post hoc analysis of five consecutive randomized clinical trials (RCTs) analyzing efficacy of anti-inflammatory medication in nondiabetic and diabetic patients undergoing routine cataract surgery.^13^^–^^17^ Study flow chart is represented in Supplementary Figure S1. Patients were enrolled between January 2016 and December 2017. Patients were admitted according to the national guidelines for the management of cataract.

Clinical outcome parameters (aqueous flare, corrected distance visual acuity, central subfield macular thickness, and intraocular pressure) were recorded at 28 days and three months, except for the RCT 2015-003296-30 in which recovery from surgery was recorded only at 28 days.^13^ We aimed to set meaningful cutoff values for aqueous flare increase that would reflect the previously agreed clinically relevant changes in the central subfield macular thickness (CSMT).^18^^,^^19^

The studies were conducted according to the tenets of the Declaration of Helsinki and were approved by the Research Director and Chief Medical Officer of the Kymenlaakso Central Hospital, the Finnish Medicines Agency Fimea and the Institutional Review Board of Helsinki University Hospital. All patients signed an informed consent before enrollment and could withdraw from the study at any time.

### Patients

All studies were conducted as randomized, prospective single-center trials (hrrg.fi/en/clinicaltrials/cataract/). Patients were randomized by a research technician for different anti-inflammatory medication protocols according to the study protocol. Patients with five reliable aqueous flare measurements preoperatively, at the day of surgery and at 28 days postoperatively were included in the study. To avoid bias from statistical dependence, in patients recruited for both eyes, only the first-operated eye was included in the post hoc analysis. Eyes with perioperative subconjunctival injection of triamcinolone acetonide were excluded from the analysis. A total of 448 eyes of 448 patients were included in the analysis.

### Anti-Inflammatory Medication

121 eyes were treated with steroid monotherapy three times a day (t.i.d.) for three weeks, 163 eyes were treated with NSAID monotherapy t.i.d. for three weeks, and 164 eyes were treated with steroid and NSAID combination therapy t.i.d. for three weeks. The length of the anti-inflammatory treatment reflected the real-life clinical practice.^20^

Steroid treatment included dexamethasone (*N* = 103, Monopex, 1 mg/ml, Laboratoires Théa, Clermont-Ferrand, France; or Oftan Dexa, 1 mg/ml, Santen Pharmaceutical Co. Ltd, Osaka, Japan) or prednisolone acetate (*N* = 18, Pred Forte, 10 mg/ml, Allergan Inc. Irvine, CA). NSAID treatment was carried out with diclofenac (*N* = 104, Voltaren Ophtha, 1 mg/ml, Laboratoires Théa; or Dicloabak, 1 mg/ml, Laboratoires Théa) or nepafenac (*N* = 59, Nevanac, 1 mg/ml, Novartis, Basel, Switzerland). Of the eyes treated with combination therapy, 118 (72%) were administered prednisolone acetate and nepafenac and 46 (28%) dexamethasone and diclofenac.

### Inclusion and Exclusion Criteria

The study subjects were aged 60 to 90 years. All enrolled patients were eligible for cataract surgery under the Current Care Guidelines of Cataract Surgery of the Finnish Medical Society Duodecim.

Exclusion criteria were prior or active wet age-related macular degeneration, retinal vein/artery occlusion, retinal detachment, retinal necrosis, vitritis/endophthalmitis, vitreous hemorrhage, retinal phlebitis or optic neuritis, myopia above −6.0 diopters, alcohol abuse, thyroid disease with abnormal thyroid-stimulating hormone (TSH) levels, chronic ocular or systemic inflammatory disorder, continuous use of anti-inflammatory drugs, known or suspected sensitivity to any of the medications used in the operation or postoperatively, and failure to use topical steroid drops. Criteria for exclusion were also intraoperative complications such as iris prolapse, use of sutures or posterior capsule tear. Diabetes mellitus was an exclusion criterion in two of the studies.^16^^,^^17^

### Surgery

Prior to the surgery, all eyes were prepared with a combination of tropicamide (Oftan Tropicamid, 5 mg/ml), phenylephrine hydrochloride (Oftan Metaoksedrin, 100 mg/ml), levofloxacin (Oftaquix, 5 mg/ml) and oxybuprocaine hydrochloride (Oftan Obucain, 4 mg/ml), all from Santen Pharmaceutical (Osaka, Japan). Povidone-iodine was applied to the periocular skin (100 mg/ml) and to the ocular surface (50 mg/ml; prepared from the Betadine 100 mg/ml, Takeda, Tokyo, Japan) for three minutes.

A standardized phacoemulsification technique was used in all cataract surgeries (hrrg.fi/en/videos/cataract/).^21^ The effect of dissipated cumulative energy (C.D.E.) and operation time (minutes) on aqueous flare were included in the analysis. Antimicrobial medication included intraoperative intracameral cefuroxime (Aprokam, Laboratoires Théa, Clermont-Ferrand, France). No postoperative antimicrobial medication was used in any of the eyes, except for one trial in which levofloxacin eye drops (Oftaquix, 5 mg/ml, Santen Pharmaceutical) were prescribed t.i.d. for one week.^13^

### Clinical Evaluation

The operating physician examined the patients preoperatively. Postoperative measurements were performed masked from the treating physicians by a trained research technician, whom the patients visited at 28 days and three months.

Refraction was evaluated with autorefractometer (ARK-1s, NIDEK Co. Ltd, Aichi, Japan). Intraocular pressure was measured by rebound tonometry (iCare tonometer, Revenio Group, Vantaa, Finland). CSMT (defined as the mean thickness in the central 1000 µm diameter area) was recorded by spectral-domain optical coherence tomography (SD-OCT; Heidelberg Eye Explorer Version 1.9.10.0 and HRA/SPECTRALIS Viewing Module Version 6.0.9.0, Heidelberg Engineering GmbH, Heidelberg, Germany) on each visit. The correct segmentation of the scans was confirmed. Follow-up 30-frame SD-OCT scans were performed with AutoRescan software.

Aqueous flare was recorded by a laser flare meter (FM-600, Kowa Company, Ltd., Nagoya, Japan) and presented as photon units/millisecond. The measurements were made before topical mydriatics or any other eye drops to avoid any possible effect on the flare values.^22^^–^^24^ Automated reliability analysis of the measurements performed by the laser flare meter was taken into account. The mean of five reliable aqueous flare measurements was used in our analysis.

The severity level of diabetic retinopathy and HbA1c value of the diabetic patients were included in the analysis.

The diagnosis of PCME was confirmed together with two retina specialists in all the studies. A 10% increase in CSMT was used as the cutoff value for macular thickening. Clinically significant PCME was defined as follows: no pre-existing macular edema on preoperative OCT, cystoid changes and macular thickening of at least 10% from the baseline in the central 1000 µm diameter area (CSMT) at any postoperative timepoint, and expected CDVA deterioration (CDVA improved less than 0.4 decimals from baseline and remained at level below 0.8 decimals) as previously described.^19^ When PCME was diagnosed, topical NSAID (nepafenac 1 mg/ml, three times a day) was prescribed for three months. Intravitreal dexamethasone implantation was recommended for PCMEs which did not respond to three months of NSAID. PCME and its treatment were not exclusion criteria.

### Statistical Analyses

Data are given as mean ± SD, except for the absolute numbers and proportions for the nominal scale. For two-group comparisons, categorical variables were analyzed with the two-factor χ² test for (or with Fisher's exact test when values in any of the cells of a contingency table were below five), continuous variables with the Student's *t*-test and nonparametric or nonnormally distributed variables with the Mann-Whitney *U* test. For multiple group comparisons, qualitative data were analyzed with the Fisher-Freeman-Halton test, continuous variables with the one-way ANOVA with Bonferroni correction and variables in ordinal measurement scale with the Kruskal-Wallis with Dunn test. A linear regression model was used to estimate the relationships between variables. Due to the positively skewed distribution of aqueous flare changes, we decided to categorize the variable as no (<50% increase from baseline), mild (50%–99%), moderate (100%–200%), and severe (>200%) increase rather than treat it as a continuous variable. IBM SPSS Statistics 25 (IBM Corp., Armonk, NY) was used for statistical analysis. *P* ≤ 0.05 was considered statistically significant.

## Results

### Baseline Variables

At 28 days, median aqueous flare change was +46.6% from the baseline (IQR from −5.1 to +131.3%) (Supplementary Figure S2 for aqueous flare change distribution). At 28 days, aqueous flare increase of at least 50% from the baseline was observed in 48.2% (216 of 448) eyes, of at least 100% from the baseline in 30.8% (138 of 448) eyes, and of at least 200% from the baseline in 12.1% (54 of 448) eyes.

Baseline patient (age, sex, diabetes), and ophthalmic variables (laterality of the eye, CDVA, CSMT, IOP) did not differ regarding the amount of aqueous flare increase from the baseline (Table 1) and regarding the proposed 100% cutoff value for aqueous flare increase (Supplementary Table S1).

**Table 1.** Baseline Variables

	Flare Increase < 50% (*N* = 232)	Flare Increase 50%–99% (*N* = 78)	Flare Increase 100%–200% (*N* = 84)	Flare Increase ≥ 200% (*N* = 54)
Sex M:F (*n*/%)	83:149 (36:64)	36:42 (46:54)	30:54 (36:64)	23:31 (43:57)
Age (y)	76.5 ± 6.8	75.8 ± 6.9	75.5 ± 6.3	75.0 ± 7.7
Laterality R:L (*n*/%)	120:112 (52:48)	47:31 (60:40)	39:45 (46:54)	22:32 (41:59)
DM (*n*/%)	79 (34)	25 (32)	26 (31)	11 (20)
Glaucoma (*n*/%)	23 (10)	7 (9)	9 (11)	5 (9)
CDVA (decimals)	0.35 ± 0.17	0.40 ± 0.18	0.37 ± 0.19	0.36 ± 0.16
IOP (mmHg)	15.0 ± 4.1	16.4 ± 4.5	15.5 ± 3.4	15.2 ± 4.2
CSMT (µm)	273.6 ± 29.6	275.6 ± 30.1	270.8 ± 24.6	266.3 ± 26.2

Data are given as mean (± SD) or absolute numbers and proportions. For multiple group comparisons, qualitative data were analyzed with the Fisher-Freeman-Halton test, continuous variables with the one-way ANOVA with Bonferroni correction and variables in ordinal measurement scale with the Kruskal-Wallis with Dunn test. CDVA; corrected distance visual acuity, CSMT; central subfield macular thickness defined as mean thickness in the central 1.0mm diameter area, DM; diabetes mellitus type I or II, IOP; intraocular pressure. Glaucoma defined by the glaucoma medication and/or treatments.

The incidences and odds ratios (ORs) for aqueous flare increase of at least 50%, 100%, and 200% among eyes receiving either steroid monotherapy, NSAID monotherapy, or a combination of both drugs are presented in Supplementary Tables S2 and S3.

Phacoemulsification energy determined as cumulative dissipated energy and operation time (in minutes) did not correlate with aqueous flare increase at 28 days (*r* = 0.044, *P =* 0.354 and *r* = 0.046, *P =* 0.324, respectively).

### Association Between Aqueous Flare and Central Subfield Macular Thickness Increases

Aqueous flare increase correlated with the changes in CSMT. At 28 days, CSMT increased 6.1 ± 32.1 µm, 12.5 ± 36.1 µm, 15.2 ± 36.7 µm, and 21.1 ± 45.8 µm in eyes with aqueous flare change <50%, or ≥50%, ≥100%, and ≥ 200% from the baseline, respectively (Table 2). At 3 months, the respective values for CSMT increase in relation to aqueous flare increase at 28 days were 0.7 ± 22.0 µm, 8.0 ± 22.9 µm, 9.5 ± 26.9 µm, and 11.0 ± 23.7 µm (Table 2). At 28 days and three months, CSMT increased significantly more among eyes with aqueous flare increase of at least 100% from the baseline when compared to those with an increase less than 100% (CSMT increase at 28 days 15.2 ± 36.7 µm vs. 6.5 ± 32.8 µm, *P* = 0.024; and at three months 9.5 ± 26.9 µm vs. 1.6 ± 20.4 µm, *P* = 0.027, Table 2).

**Table 2.** Macular Thickness Change in Relation to Aqueous Flare Increase at 28 Days

Flare Increase (from Baseline)	CSMT Change (µm) at 28 Days	CSMT Change (µm) at 3 Months
<50% (*N* = 232)	+6.1 ± 32.1	+0.7 ± 22.0
≥50% (*N* = 216)	+12.5 ± 36.1	+8.0 ± 22.9
*P* =	0.077	0.022^†^
<100% (*N* = 310)	+6.5 ± 32.8	+1.6 ± 20.4
≥100% (*N* = 138)	+15.2 ± 36.7	+9.5 ± 26.9
*P* =	0.024^†^	0.027^†^
<200% (*N* = 394)	+7.6 ± 35.5	+2.6 ± 22.4
≥200% (*N* = 54)	+21.1 ± 45.8	+11.0 ± 23.7
*P* =	0.072	0.267

Data are given as mean ± SD. For two-group comparisons, continuous variables were analyzed with the Student's *t*-test. CSMT; central subfield macular thickness.

† *P* < 0.05.

At 28 days, macular thickening (at least a 10% CSMT increase from the baseline) was observed in 6.0%, 6.5%, 9.4%, and 13.0% of eyes with <50%, or ≥50%, ≥100%, and ≥200% aqueous flare increase from the baseline (Table 3). At three months, the respective incidences in relation to aqueous flare increase were 4.9%, 9.4%, 12.7%, and 17.4% (Table 3).

**Table 3.** Macular Thickening Incidence in Relation to Aqueous Flare Increase at 28 Days

Flare Increase	<50%	≥50%	*P* =
CSMT increase ≥ 10%			
at 28 days	6.0% (14/232)	6.5% (14/216)	0.845
at 3 months	4.9% (6/123)	9.4% (8/85)	0.182
Flare increase	<100%	≥100%	
CSMT increase ≥ 10%			
at 28 days	4.8% (15/310)	9.4% (13/138)	0.064
at 3 months	4.6% (7/153)	12.7% (7/55)	0.033^†^
Flare increase	<200%	≥200%	
CSMT increase ≥ 10%			
at 28 days	5.3% (21/394)	13.0% (7/54)	0.030^†^
at 3 months	5.4% (10/185)	17.4% (4/23)	0.048^†^

Data are given as proportions. For two-group comparisons, qualitative data were analyzed with the two-factor χ² test. CSMT; central subfield macular thickness.

† *P* < 0.05.

In eyes that developed PCME (*N* = 13), aqueous flare increased 146.9 ± 131.0% (median and IQR; 131.5%, 42.8%–215.3%) at 28 days, compared to 95.5 ± 225.5% (median and IQR; 44.4%, −5.4% to +127.4%) in eyes without PCME (*N* = 435) (*P* = 0.016, Supplementary Table S4). Odds ratios for macular thickening and PCME according to aqueous flare change are presented in Table 4.

**Table 4.** Odds Ratios for Clinical Endpoints in Relation to Aqueous Flare Increase at 28 Days

Flare Increase	≥50% (Cohort < 50%)	≥100% (Cohort < 100%)	≥200% (Cohort < 200%)
CSMT increase ≥ 10% at 28 days	OR 0.995	OR 1.930	OR 2.735
	95CI (0.450−2.201)	95CI (0.867−4.299)	95CI (1.089−6.867)
	*P* = 0.990	*P* = 0.102	*P* = 0.026
CSMT increase ≥ 30% at 28 days	OR 1.507	OR 2.198	OR 3.026
	95CI (0.419–5.423)	95CI (0.625–7.730)	95CI (0.757–12.092)
	*P* = 0.527	*P* = 0.209	*P* = 0.100
BCVA no gain at 28 days	OR 2.913	OR 1.867	OR 2.618
	95CI (0.762–11.127)	95CI (0.560–6.226)	95CI (0.673–10.177)
	*P* = 0.102	*P* = 0.302	*P* = 0.150
PCME	OR 1.714	OR 1.968	OR 2.117
	95CI (0.552–5.323)	95CI (0.649–5.967)	95CI (0.564–7.940)
	*P* = 0.346	*P* = 0.224	*P* = 0.256

BCVA; best-corrected visual acuity, CSMT; central subfield macular thickness, PCME; pseudophakic cystoid macular edema.

### Association Between Aqueous Flare and Central Subfield Macular Thickness Increases in Diabetic and Nondiabetic Patients and Eyes With and Without Pseudoexfoliation

Baseline patient (age), ophthalmic (CDVA, CSMT), and surgical variables (operation time, phacoemulsification energy) did not differ between nondiabetic and diabetic patients (Supplementary Table S5). Diabetic patients had mean serum glycosylated hemoglobin [HbA1c] 51.4 ± 14.8 mmol/mol (6.85 ± 1.36%), higher male distribution (45% vs. 35% males, *P* = 0.039, Supplementary Table S5), and higher aqueous flare levels (12.9 ± 11.8 vs. 9.8 ± 8.2 photon units/ms, *P* < 0.001, Supplementary Table S5) when compared to nondiabetic patients. In eyes with pseudoexfoliation, higher female distribution (74% vs. 58% females, *P* = 0.003, Supplementary Table S5), and higher patient age (77.8 ± 5.9 vs. 75.4 ± 7.0 years, *P* = 0.002, Supplementary Table S5) were observed when compared to eyes without pseudoexfoliation. Ophthalmic and surgical variables did not differ between those with and without pseudoexfoliation (Supplementary Table S5).

At 28 days the proportions of eyes with aqueous flare increase < 50%, or ≥ 50%, ≥ 100%, and ≥ 200% from the baseline were comparable between nondiabetic and diabetic patients (Supplementary Table S6). In pseudoexfoliation eyes, at 28 days the proportions of eyes with aqueous flare increase ≥200% from the baseline was significantly higher when compared to eyes without pseudoexfoliation (Supplementary Table S6).

## Discussion

Here, we provide more insight into blood-aqueous-barrier (BAB) disruption after cataract surgery and the association between aqueous flare increase and macular thickening among nondiabetic and diabetic patients. We were able to discover an association between aqueous flare increase, macular thickening, and PCME. Diabetic patients presented higher aqueous flare levels at baseline than nondiabetic patients, but comparable postoperative proportions in aqueous flare increase were observed between the groups. Importantly, a 100% cutoff value for aqueous flare increase at 28 days could potentially be used when studying the anti-inflammatory efficacy of different treatment protocols and determining the indication for extending anti-inflammatory therapy.

Aqueous flare is a good indicator of an intraocular inflammatory process. A laser flare meter is recognized as easy to operate, noninvasive, and more precise compared to fluorophotometry.^25^^,^^26^ Flare values have been shown to alternate according to changes in the blood-retina-barrier (BRB) integrity in retinal diseases, ^12^^,^^27^^,^^28^ and they correlate well with the amount of leakage in fluorescein angiography.^29^ Prolonged inflammation and cytokine presentation predispose operated eyes to early and late complications after surgery.^8^ A connection between ocular inflammation and PCME has been recognized as these patients had significantly higher flare values compared to pseudophakic controls.^7^ Increased aqueous flare has been associated with a profibrotic cytokine environment.^30^ Furthermore, a connection between aqueous flare levels and the development of postoperative proliferative vitreoretinopathy has been identified.^31^ Interestingly, the preoperative aqueous flare values ranged considerably between the cataract surgery patients. Relative changes in aqueous flare compared to the absolute aqueous flare values seemed to better reflect the BAB breakdown induced macular changes postoperatively.

Flare levels were shown to be higher and remain elevated after cataract surgery in diabetic patients with clinically significant macular edema compared to those with nonproliferative diabetic retinopathy and patients without diabetes.^32^^,^^33^ On the other hand, systemic vasoactive medications were found to be overrepresented in diabetic patients, possibly having a bearing on the incidence of PCME.^15^ When diabetes was appropriately managed, eyes without posterior segment manifestations were not at increased risk for PCME.^34^ Here, in diabetic patients with predominately none or mild background retinopathy, the proportions of eyes with aqueous flare increase were comparable to nondiabetic patients. In addition, macular thickness increase across the groups with varying amounts of BAB disruption was even more modest in diabetic than in nondiabetic patients.

During the course of anti-inflammatory medication, aqueous flare is expected to recover to near-normal values. Here we aimed to pick up prolonged inflammation after the course of topical anti-inflammatory treatment at the time of routine checkup of cataract surgery patients. Leftover ophthalmic viscosurgical device (OVD) and even topical postoperative eye drops in a similar fashion to mydriatics may compromise flare measurement reliability.^21^^–^^23^ Thus, the 28-day postoperative timepoint of otherwise uneventful surgeries may provide a time window for reliable measurement of prolonged inflammation with minimal confounders arising from the surgical procedure in itself. Among several identified risk factors for the development of PCME,^1^^,^^13^^,^^18^^,^ ^35^^–^^37^ aqueous flare increase at 28 days of at least 100% from baseline was associated with macular thickening. In clinical practice flare values exceeding this cutoff value could be considered as an indication for extending anti-inflammatory therapy. Eyes with perioperative subconjunctival injection of triamcinolone acetonide (TA) were excluded from the analyses. Subconjunctival TA depot provides a treatment method with prolonged anti-inflammatory therapeutic effect which can also reduce the complications related to patient nonadherence with eye drop administration. Pharmacologically active TA has been identified even up to 13 months following subconjunctival injection.^38^ Since the anti-inflammatory kinetics of TA differs greatly from the drops, and the analyses were based on the 28-day aqueous flare change, we decided to include only those cataract surgery patients with post-operative anti-inflammatory eye drops.

Our study has some limitations that should be taken into consideration. First, this data is presented as a post hoc analysis gathered from five individual consecutive RCTs. However, all the trials were conducted with the same standardized cataract surgery protocol having special emphasis on the assessment of changes in aqueous flare and macular thickness. When interpreting the findings of the study, caution must be taken to generalize our findings to different anti-inflammatory regimens such as tapering down with longer treatment. Moreover, aqueous flare changes with and without preoperative anti-inflammatory medication deserves to be further studied. Second, the anti-inflammatory medication depended on the study design of an individual RCT. The effect of topical NSAIDs as a monotherapy or in combination with topical steroids in reducing the risk of PCME has been previously studied.^13^^,^^18^^,^^36^ Patients treated with a combination of topical steroids and NSAIDs had a lower risk for developing PCME than patients treated with either of the two drugs solely.^18^ Our findings emphasize that diminished aqueous flare changes after cataract surgery when administering topical NSAIDs compared to steroid monotherapy may involve the mechanism of PCME prevention. Nevertheless, this study was not designed to compare the efficacy of steroids, NSAIDs, or their combination against BAB disruption. Third, simple and robust statistical models were used to estimate association between aqueous flare and macular changes. The rationale for selecting robust models was our assumption that they would result in predictions better fitting to the noise to minimize false estimations when compared to more complex prediction models better suitable for large registries and meta-analyses. Fourth, the baseline CSMT tended to differ between the groups. Thus, despite the significant changes in CSMT at postoperative timepoints, it may not reflect the absolute CSMT values. Fifth, we could not prove significant correlations between aqueous flare changes with functional visual measurements, for example, visual acuity. Thus, the clinical relevance of aqueous flare changes on functional measurement endpoints yet needs to be addressed. It should also be recognized that the study designs of the RCTs were aligned to investigate the efficacy of anti-inflammatory medication for cataract surgery considering that PCME is often observed within 4 to 12 weeks after cataract surgery. Thus, we lack long-term follow-up beyond the three months. Previously, it was pointed out that macular changes remain long-lasting and refractory in a minority of patients.^19^^,^^39^

In a clinical setting, aqueous flare measurements by well-trained technicians and ophthalmic nurses may offer fast, noninvasive and repeatable method supplementing the physician's estimation of BAB disruption and a variable for effective analytics decision making. We could not set a clear cutoff value for PCME, as the mechanisms seem multifactorial. Combining aqueous flare changes with other patient-related characteristics, using more sophisticated algorithms^40^ and AI would be better predict the BAB disruption in early and late recovery from surgery, PCME risk, and provide treatment strategies in clinical practice. Identifying patients more vulnerable for the development of PCME using flare measurement may provide better patient care and pre-emptive surgical or medical consideration.

## Supplementary Material

Supplement 1

Supplement 2

Supplement 3

Supplement 4

Supplement 5

Supplement 6

Supplement 7

Supplement 8
