# Supplementary material for: Relationship Between Prolonged Intraocular Inflammation and Macular Edema After Cataract Surgery
Source: Transl Vis Sci Technol. 2021 Jun 14;10(7):15. doi: 10.1167/tvst.10.7.15 (PMC8212433; doi:10.1167/tvst.10.7.15)
Supplement: Supplement 1 [file tvst-10-7-15_s001.pdf]

Supplement Figure 1

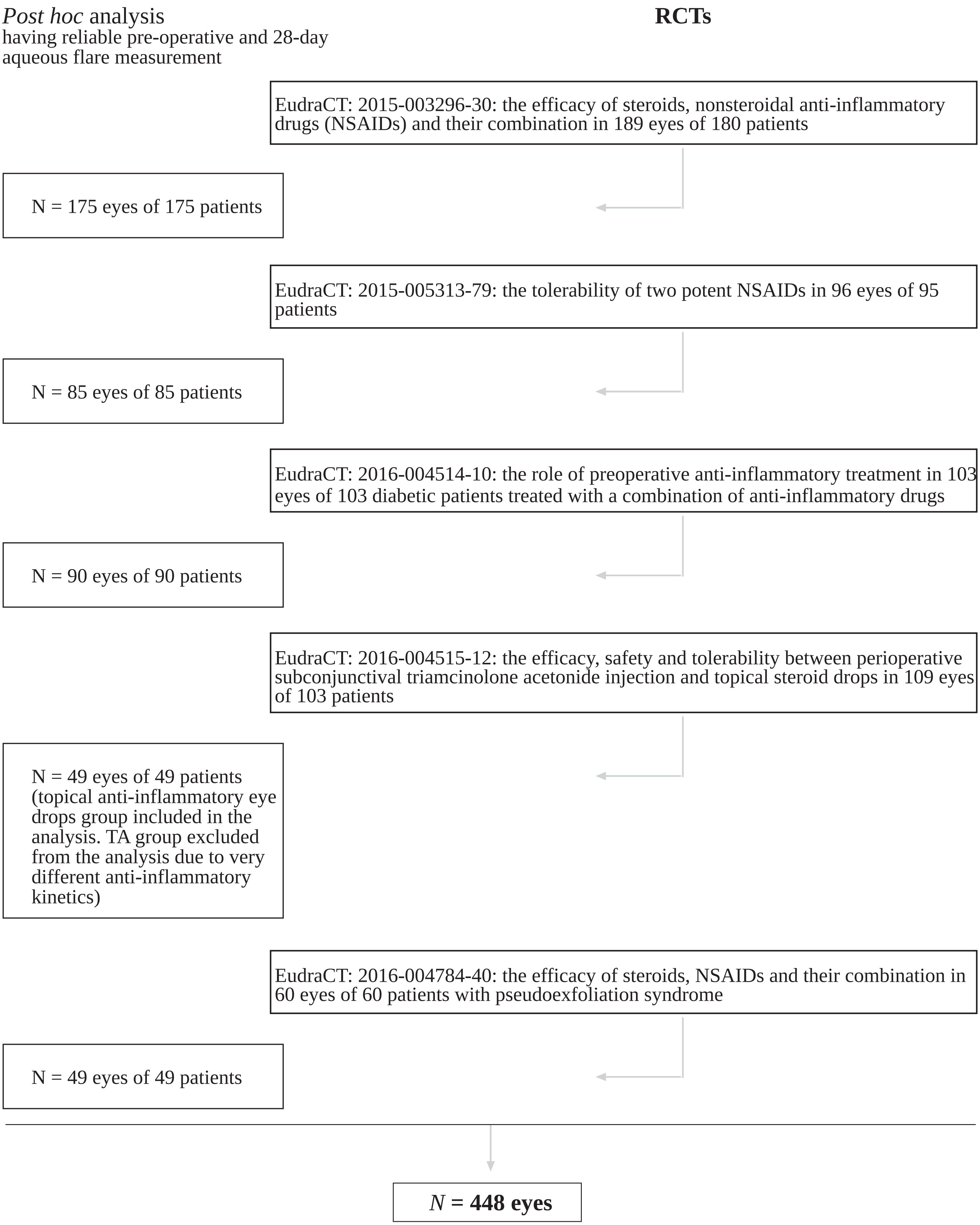

Supplement Figure 1. *Study flow chart.*

A *post hoc* analysis of five consecutive randomized clinical trials (RCTs) analyzing efficacy of anti-inflammatory medication in non-diabetic and diabetic patients undergoing routine cataract surgery.
