# Supplementary material for: Relationship Between Prolonged Intraocular Inflammation and Macular Edema After Cataract Surgery
Source: Transl Vis Sci Technol. 2021 Jun 14;10(7):15. doi: 10.1167/tvst.10.7.15 (PMC8212433; doi:10.1167/tvst.10.7.15)
Supplement: Supplement 2 [file tvst-10-7-15_s002.pdf]

Supplement Figure 2

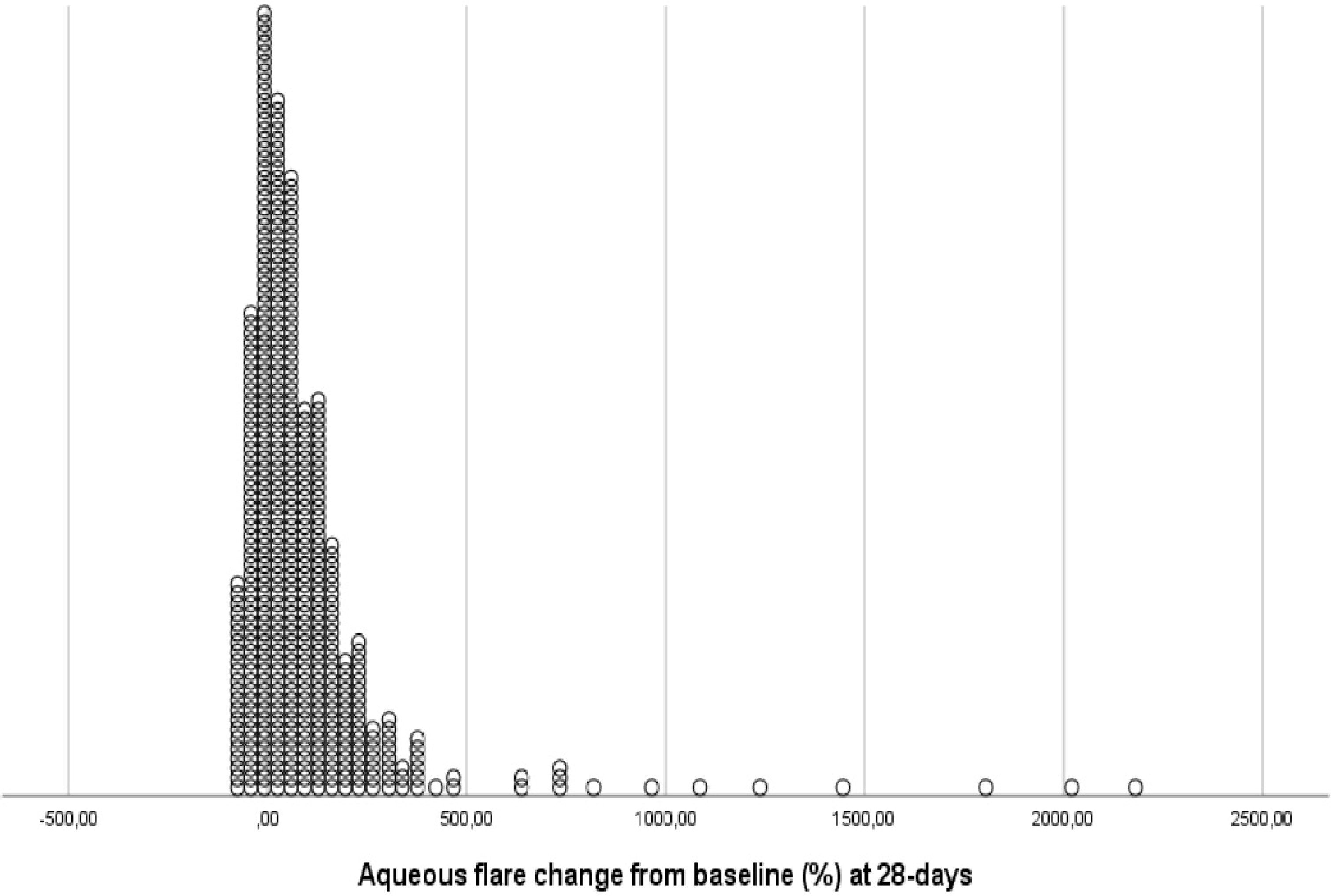

Supplement Figure 2. *Aqueous flare change distribution at 28 days.*
Aqueous flare levels (photon units / msec) were recorded before surgery and after the course of topical anti-inflammatory treatment at 28 days.
