# Supplementary material for: Relationship Between Prolonged Intraocular Inflammation and Macular Edema After Cataract Surgery
Source: Transl Vis Sci Technol. 2021 Jun 14;10(7):15. doi: 10.1167/tvst.10.7.15 (PMC8212433; doi:10.1167/tvst.10.7.15)
Supplement: Supplement 3 [file tvst-10-7-15_s003.pdf]

Supplement Table 1. Baseline variables

	**Flare increase < 100% (*N* = 310)**	**Flare increase ≥ 100% (*N* = 138)**	***P* =**
Sex M:F (n/%)	119:191 (38:62)	53:85 (38:62)	0.997
Age (y)	76.3 ± 6.8	75.3 ± 6.9	0.195
Laterality R:L (n/%)	167:143 (54:46)	61:77 (44:56)	0.588
DM (n/%)	104 (34)	37 (27)	0.156
Glaucoma (n/%)	30 (10)	14 (10)	0.878
CDVA (decimals)	0.36 ± 0.18	0.36 ± 0.17	0.825
IOP (mmHg)	15.4 ± 4.2	15.4 ± 3.7	0.995
CSMT (µm)	274.4 ± 29.0	269.4 ± 25.5	0.086

Data are given as mean (±SD) or absolute numbers and proportions. For two-group comparisons, qualitative data were analyzed with the two-factor χ^2 test, continuous variables with the Student's T test and variables in ordinal measurement scale with the Mann-Whitney U test. CDVA; corrected distance visual acuity, CSMT; central subfield macular thickness defined as mean thickness in the central 1.0mm diameter area, DM; diabetes mellitus type I or II, IOP; intraocular pressure. Glaucoma defined by the glaucoma medication and/or treatments.
