# Supplementary material for: Relationship Between Prolonged Intraocular Inflammation and Macular Edema After Cataract Surgery
Source: Transl Vis Sci Technol. 2021 Jun 14;10(7):15. doi: 10.1167/tvst.10.7.15 (PMC8212433; doi:10.1167/tvst.10.7.15)
Supplement: Supplement 4 [file tvst-10-7-15_s004.pdf]

Supplement Table 2. Incidences of aqueous flare increase at 28 days

Change from baseline	N =	Incidence (%)
Steroid (N=121)		
Flare increase < 50%	49	40.5
Flare increase 50 - 99%	18	14.9
Flare increase 100 - 200%	31	25.6
Flare increase ≥ 200%	23	19.0
NSAID (N=163)		
Flare increase < 50%	95	58.3
Flare increase 50 - 99%	30	18.4
Flare increase 100 - 200%	24	14.7
Flare increase ≥ 200%	14	8.6
Steroid + NSAID (N=164)		
Flare increase < 50%	88	53.7
Flare increase 50 - 99%	30	18.3
Flare increase 100 - 200%	29	17.7
Flare increase ≥ 200%	17	10.4

Data are given as absolute numbers and proportions. NSAID; non-steroidal anti-inflammatory drug
