# Supplementary material for: Relationship Between Prolonged Intraocular Inflammation and Macular Edema After Cataract Surgery
Source: Transl Vis Sci Technol. 2021 Jun 14;10(7):15. doi: 10.1167/tvst.10.7.15 (PMC8212433; doi:10.1167/tvst.10.7.15)
Supplement: Supplement 5 [file tvst-10-7-15_s005.pdf]

Supplement Table 3. Odds ratios for aqueous flare increase in relation to anti-inflammatory medication

**Anti-inflammatory medication**	**NSAID** (cohort steroid)	**NSAID + steroid** (cohort steroid)
Aqueous flare increase **≥ 50%**	OR 0.490<br>95CI 0.306-0.783<br>*P*=0.004	OR 0.578<br>95CI 0.359-0.932<br>*P*=0.033
Aqueous flare increase **≥ 100%**	OR 0.392<br>95CI 0.238-0.644<br>*P*<0.001	OR 0.458<br>95CI 0.278-0.755<br>*P*=0.003
Aqueous flare increase **≥ 200%**	OR 0.416<br>95CI 0.211-0.817<br>*P*=0.015	OR 0.455<br>95CI 0.228-0.907<br>*P*=0.036

Aqueous flare increase (%) at 28 days represented in relation to preoperative value.
