# Supplementary material for: Relationship Between Prolonged Intraocular Inflammation and Macular Edema After Cataract Surgery
Source: Transl Vis Sci Technol. 2021 Jun 14;10(7):15. doi: 10.1167/tvst.10.7.15 (PMC8212433; doi:10.1167/tvst.10.7.15)
Supplement: Supplement 6 [file tvst-10-7-15_s006.pdf]

Supplement Table 4. Aqueous flare in eyes with and without PCME

	**PCME –** (N=435)	**PCME +** (N=13)	***P* =**
Preoperatively (pu/ms)	10.7 ± 9.3	9.7 ± 5.0	0.783
**Aqueous flare change (%)**			
At 28 days	+95.5 ± 225.5 (44.4, -5.4 – 127.4)	+146.9 ± 131.0 (131.5, 42.8 – 215.3)	0.016†

Data are given as mean ±SD (and median with IQR). For two-group comparisons, variables were analyzed with the non-parametric Mann-Whitney U test. Clinically-significant PCME was defined as follows: no pre-existing macular edema on preoperative OCT, cystoid changes and macular thickening of at least 10% from the baseline in the central 1000-μm diameter area (CSMT) at any postoperative time-point, and expected CDVA deterioration (CDVA improved less than 0.4 decimals from baseline and remained at level below 0.8 decimals). CDVA; corrected distance visual acuity, CSMT; central subfield macular thickness, PCME; pseudophakic cystoid macular edema, pu; photon units. †$P<0.05$
