# Supplementary material for: Relationship Between Prolonged Intraocular Inflammation and Macular Edema After Cataract Surgery
Source: Transl Vis Sci Technol. 2021 Jun 14;10(7):15. doi: 10.1167/tvst.10.7.15 (PMC8212433; doi:10.1167/tvst.10.7.15)
Supplement: Supplement 7 [file tvst-10-7-15_s007.pdf]

Supplement Table 5. Baseline variables in diabetic and non-diabetic patients and eyes with and without pseudoexfoliation

	**DM -** (N=307)	**DM +** (N=141)	***P* =**	**PXF –** (N=341)	**PXF +** (N=107)	***P* =**
Gender M:F (n/%)	108:199 (35:65)	64:77 (45:55)	0.039[†]	144:197 (42:58)	28:79 (26:74)	0.003[†]
Age (y)	76.1 ± 6.7	76.7 ± 7.2	0.432	75.4 ± 7.0	77.8 ± 5.9	0.002[†]
DR (No:BG:NPDR:PDR)		88:16:4:6				
Aqueous flare (pu/msec)	9.8 ± 8.2	12.9 ± 11.8	<0.001[†]	10.4 ± 9.7	11.9 ± 9.3	0.147
CDVA (decimals)	0.36 ± 0.17	0.36 ± 0.18	0.650	0.36 ± 0.18	0.36 ± 0.17	0.836
CSMT (µm)	272.0 ± 31.0	277.4 ± 31.0	0.076	274.5 ± 30.8	270.0 ± 32.7	0.179
Operation time (min)	19.6 ± 9.4	18.8 ± 9.3	0.326	19.6 ± 9.3	18.9 ± 9.5	0.464
Phaco energy (CDE)	20.3 ± 11.2	20.1 ± 11.1	0.875	19.9 ± 11.2	20.9 ± 10.9	0.365

Data are given as mean (±SD) or absolute numbers and proportions. For two-group comparisons, qualitative data were analyzed with the two-factor χ^2 test, continuous variables with the Student's T test and variables in ordinal measurement scale with the Mann-Whitney U test. BG; mild to moderate background diabetic retinopathy, CDE; cumulative dissipated energy, CDVA; corrected distance visual acuity, CSMT; central subfield macular thickness, DM; diabetes mellitus type I or II, NPRD; severe nonproliferative (preproliferative) diabetic retinopathy, PDR; proliferative diabetic retinopathy. [†]$P<0.05$
