# Supplementary material for: Relationship Between Prolonged Intraocular Inflammation and Macular Edema After Cataract Surgery
Source: Transl Vis Sci Technol. 2021 Jun 14;10(7):15. doi: 10.1167/tvst.10.7.15 (PMC8212433; doi:10.1167/tvst.10.7.15)
Supplement: Supplement 8 [file tvst-10-7-15_s008.pdf]

Supplement Table 6. Aqueous flare increase in patients with diabetes and eyes with pseudoexfoliation

	**DM –** (N=307)	**DM +** (N=141)	***P* =**	**PXF –** (N=341)	**PXF +** (N=107)	***P* =**
**Aqueous flare increase**						
< 50% (N/%)	153 (50)	79 (56)		183 (54)	49 (46)	
≥ 50% (N/%)	154 (50)	62 (44)	0.223	158 (46)	58 (54)	0.155
≥ 100% (N/%)	101 (33)	37 (26)	0.156	100 (29)	38 (36)	0.226
≥ 200% (N/%)	43 (14)	11 (8)	0.061	35 (10)	19 (18)	0.038†

Data are given as absolute numbers and proportions or mean ± SD. For two-group comparisons, qualitative data were analyzed with the two-factor χ^2 test. CSMT; central subfield macular thickness, DM; diabetes mellitus type I or II, PXF, pseudoexfoliation. †$P<0.05$.
